# Supplementary figures and images for: An entropic safety catch controls hepatitis C virus entry and antibody resistance
Source: eLife. 2022 Jul 7;11:e71854. doi: 10.7554/eLife.71854 (PMC9333995; doi:10.7554/eLife.71854)

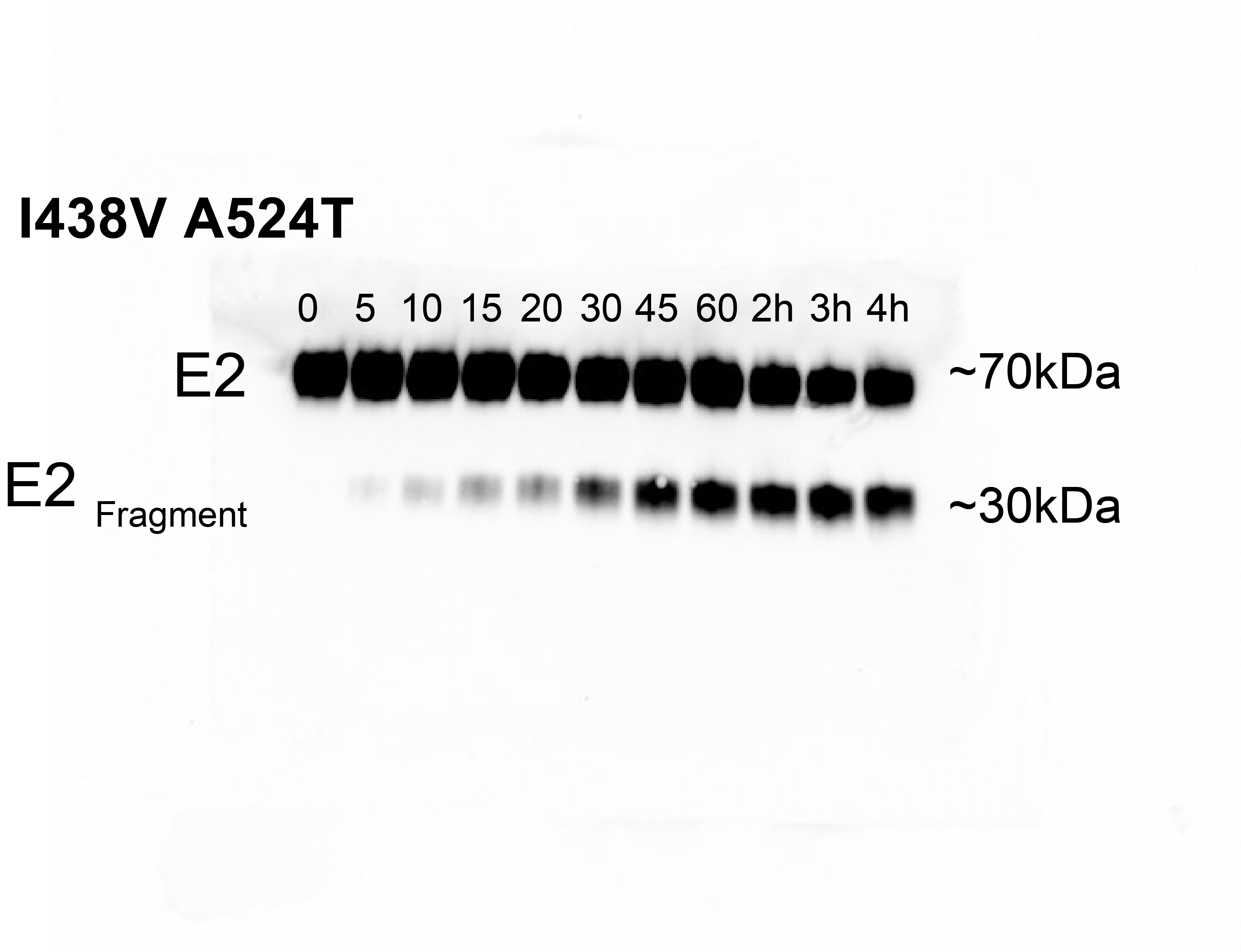

Supplement: Figure 4—figure supplement 1—source data 1. [file elife-71854-fig4-figsupp1-data1.zip › Figure 4 - figure supplement 1 source data/Figure 4 - figure supplement 1 source data D.jpg]

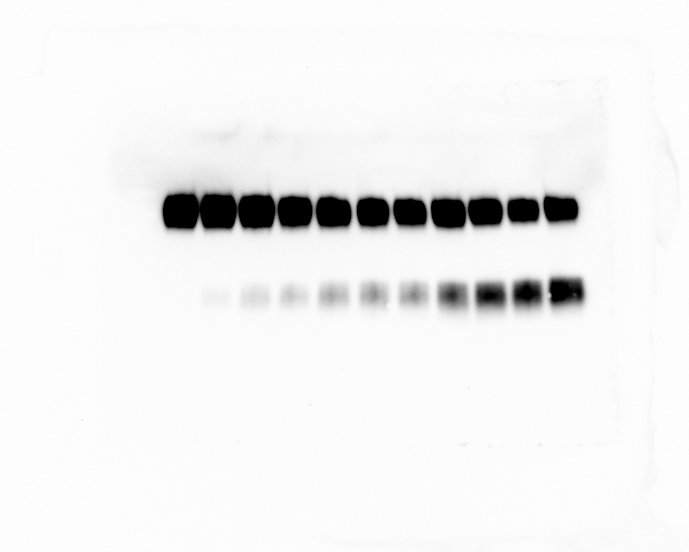

Supplement: Figure 4—figure supplement 1—source data 1. [file elife-71854-fig4-figsupp1-data1.zip › Figure 4 - figure supplement 1 source data/Figure 4 - figure supplement 1 source data A.jpg]

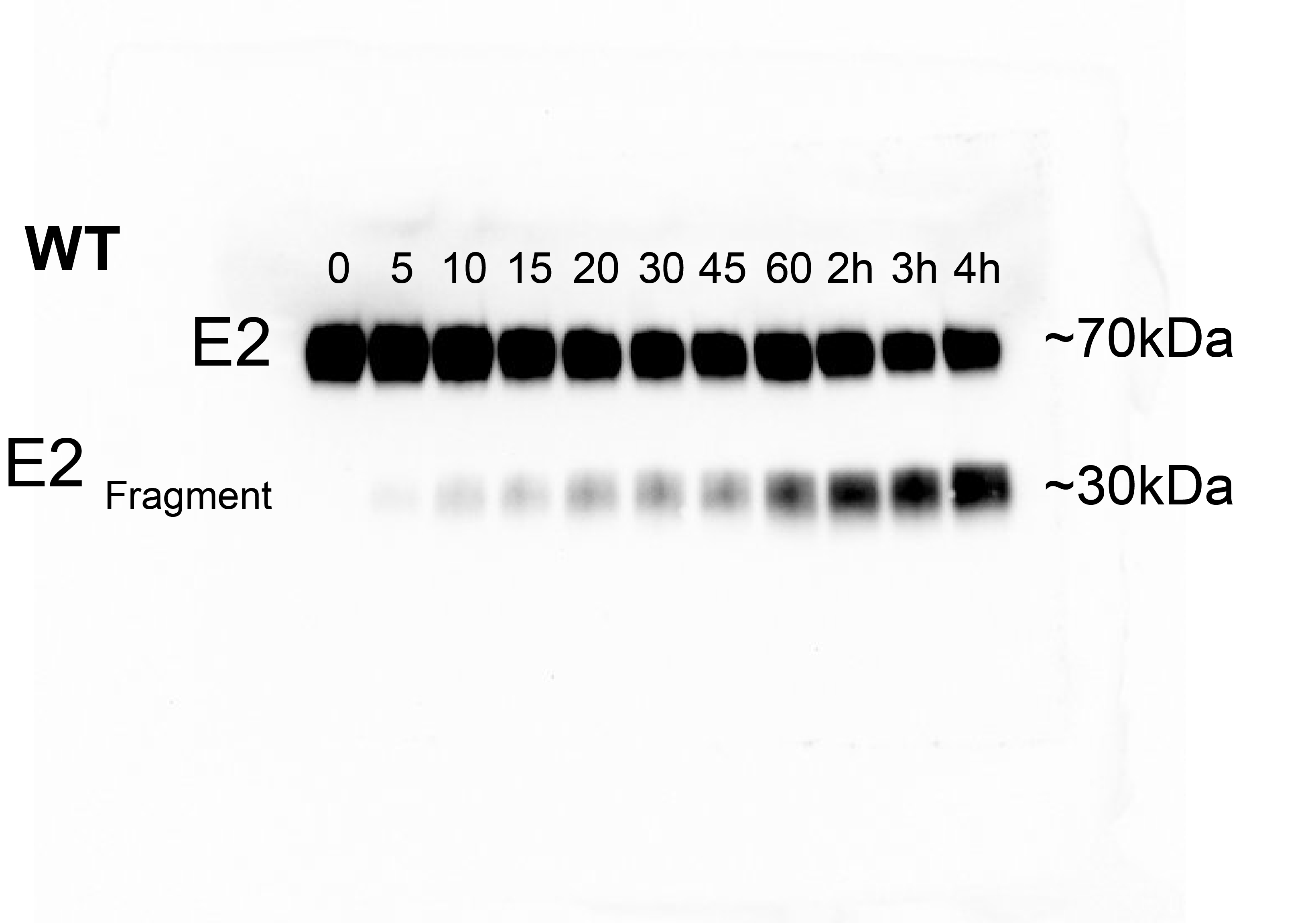

Supplement: Figure 4—figure supplement 1—source data 1. [file elife-71854-fig4-figsupp1-data1.zip › Figure 4 - figure supplement 1 source data/Figure 4 - figure supplement 1 source data B.jpg]

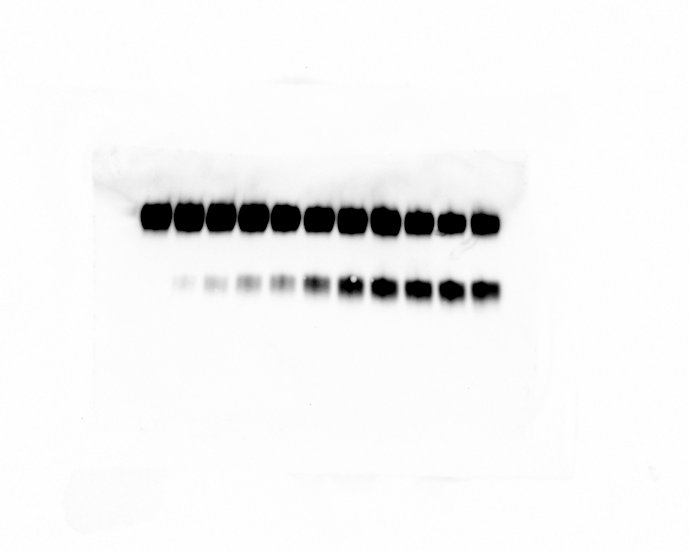

Supplement: Figure 4—figure supplement 1—source data 1. [file elife-71854-fig4-figsupp1-data1.zip › Figure 4 - figure supplement 1 source data/Figure 4 - figure supplement 1 source data C.jpg]
